# Assessment of thyroid gland hormones and ultrasonographic abnormalities in medical staff occupationally exposed to ionizing radiation

**DOI:** 10.1186/s12902-022-01196-z

**Published:** 2022-11-21

**Authors:** Sanaa A. El-Benhawy, Enayat I. Fahmy, Sherien M. Mahdy, Galal H. Khedr, Alyaa S. Sarhan, Mohamed H. Nafady, Yousef A. Yousef Selim, Tarek M. Salem, Nehal Abu-Samra, Hany A. El Khadry

**Affiliations:** 1 Radiation Sciences Department, Medical Research Institute, Alexandria University, Alexandria, Egypt; 2 Nuclear Medicine and thyroid gland Department, Naser Institute for Research and Treatment, Nasr City, Egypt; 3 Radiology and Medical Imaging, Faculty of Applied Medical Sciences, Misr University for Science & Technology, Giza, Egypt; 4 Radiology and Medical Imaging, Faculty of Applied Medical Sciences, University of 6 October, Giza, Egypt; 5 Department of Internal Medicine, (Endocrinology Unit), Faculty of Medicine, Alexandria University, Alexandria, Egypt; 6 Department of Basic Sciences, Faculty of Physical Therapy, Pharos University, Alexandria, Egypt; 7 Applied Medical Chemistry Department, Medical Research Institute, Alexandria University, Alexandria, Egypt

**Keywords:** Ionizing radiation, Thyroid gland, Radioiodine, X-ray, Ultrasound

## Abstract

**Background:**

Ionizing radiation (IR) is high-energy radiation that has the potential to displace electrons from atoms and break chemical bonds. It has the ability to introduce mutations, DNA strand breakage, and cell death. Being a radiosensitive organ, exposure of the thyroid gland to IR can lead to significant changes in its function.

**Aim of the work:**

Was to measure the levels of thyroid hormones panel and ultrasonography abnormalities in medical staff occupationally exposed to IR.

**Subjects and methods:**

A total of 120 subjects were divided into three main groups: Group I: radiation-exposed workers occupationally exposed to radioiodine (131I) (*n* = 40), Group II: radiation-exposed workers occupationally exposed to X-ray (*n* = 40), and Group III: non-exposed healthy professionals matched in age and sex with the previous groups (*n* = 40). Thyroid hormones panel including free triiodothyronine (fT3), free thyroxine (fT4), thyroid-stimulating hormone (TSH), anti-thyroperoxidase antibodies (anti-TPO), and thyroglobulin (Tg) were measured. Thyroid ultrasonography was performed. Oxidative stress markers such as malondialdehyde (MDA), hydrogen peroxide (H2O2), and total antioxidant capacity (TAC) were measured.

**Results:**

Group I had significantly higher fT3 levels than the control group. fT3 levels were considerably higher, while TSH was substantially lower in group II participants than in the control group. Tg was markedly lower in radiation-exposed workers. However, anti-TPO levels in radiation-exposed workers were significantly higher than in the control group. MDA and H_2_O_2_ were substantially higher; TAC was significantly lower in radiation-exposed workers compared to the control group. According to ultrasonographic examination, thyroid volume and the percentage of thyroid nodules in all radiation workers were significantly higher than in the control group.

**Conclusion:**

Despite low exposure doses, occupational exposure to IR affects the thyroid hormones and links with a higher likelihood of developing thyroid immune diseases.

## Background

Ionizing radiation (IR) is energy in the form of waves or particles that knocks atoms’ electrons out of place. It is capable of causing DNA strand breaks and mutations. IR is mostly genotoxic agent and a known carcinogen. Ionizing radiation’s impact on human health has been thoroughly documented throughout the past century. There is a growing evidence from researches indicating the association between IR and cancer. It is best represented by a linear nonthreshold model [1, 2]. There is a general agreement that exposure to large doses of IR poses a serious risk to human health. On the other hand, numerous scientists have voiced rising scepticism and put up various hypotheses on the dangers associated with long-term exposures to low doses of IR, which occur more frequently than exposure to large doses [3].

Ionizing radiation has become a necessary component of modern life, particularly in the medical field. In other circumstances, it is utilized for both diagnostic and therapeutic purposes, such as cancer. Radiation workers are exposed to IR in the workplace. With developments in modern medicine, radio-diagnosis and radiotherapy are being utilised more frequently which lead to an increase in the number of occupationally exposed persons [4].

Our previous findings revealed that, workers occupationally exposed to low doses of IR showed higher incidence of all types of chromosomal aberrations and elevated levels of serum 8-OHdG [5]. Moreover, Occupational exposure to IR alters circulating redox and inflammatory biomarkers [6]. Recently, significant increase in methemoglobin levels and significant decrease in MCV and ferritin levels were found among radiation-exposed workers [7].

The thyroid gland, which receives a significant radiation dosage from scatter radiation due to its anatomical location, is one of the target organs for radiation-related disease [8, 9]. The thyroid gland is the largest endocrine gland, with two lobes beside the trachea and a lower larynx. The thyroid gland produces hormones and regulates basal metabolic rate, protein synthesis, and several other processes, including development. Iodine and tyrosine make thyroid hormones T3 and T4 in follicular cells. Calcitonin hormone is produced by the thyroid and is involved in calcium homeostasis [10]. While the relation between thyroid irradiation and an increased risk of thyroid cancer is well known, the effects of radiation on thyroid gland function have received less attention [11]. Low-dose radiation’s effects on thyroid hormone levels have only been studied in few studies [12, 13].

Radiation causes oxidative stress, which happens when there is an imbalance between reactive oxygen species (ROS) and antioxidants. Cells boost defensive enzymes and proteins to counteract the oxidant property and redox balance [14–16]. Although oxidative reactions occur in all tissues and organs, oxidative activities are essential for thyroid hormone synthesis in the thyroid gland. Under normal conditions, the thyroid creates substantial ROS, mainly hydrogen peroxide (H_2_O_2_) [17]. On the other hand, increased oxidative stress caused by ionizing radiation causes more damage to macromolecules, potentially leading to thyroid problems and cancer. Free radicals including hydroxyl, superoxide, nitric oxide, and hydrogen peroxide radicals are produced by ionizing radiation. These free radicals are chemically very active acting as oxidizing agents causing morphological and physiological changes in the cells. In thyroid gland cells, these radicals have the potential to interact with other macromolecules in thyroid cells and alter their structure and function, leading to hypo- or hyperthyroid disorders [2]. Therefore, the present study aimed to investigate whether exposed medical personnel are more likely to develop thyroid hormones and gland abnormalities. To achieve this goal, a thyroid hormones panel including free triiodothyronine (fT3), free thyroxine (fT4), thyroid-stimulating hormone (TSH), anti-thyroperoxidase antibodies (anti-TPO), and thyroglobulin (Tg) were measured in the serum samples of all studied groups. In addition, oxidative stress markers such as malondialdehyde (MDA), hydrogen peroxide (H_2_O_2_), and total antioxidant capacity (TAC) were assayed.

## Methods

The present prospective study included a total of 120 subjects divided into three main groups: Group I: radiation-exposed workers occupationally exposed to radioactive Iodine-131(*n* = 40). Group II: radiation-exposed workers occupationally exposed to X-ray (*n* = 40). Group III: non-exposed healthy professionals matched in age and sex with both groups (*n* = 40), in the period between April 2019 till January 2020. Group I participants were selected from Nuclear Medicine Department, while group II participants were selected from Diagnostic Radiology and Radiotherapy Departments, National Cancer Institute, Cairo University, Egypt.. The Group III participants were health professionals selected from the different departments not exposed to ionizing radiation. After approval of The Ethical Committee of the Medical Research Institute, Alexandria University, Alexandria, Egypt, on the protocol of the present study, informed consent was taken from every participant. The study was done according to The Code of Ethics of the World Medical Association (Declaration of Helsinki) for studies involving humans. All subjects were interviewed and completed a questionnaire including demographic data, lifestyles, medical records and radiation exposure history. The radiation workers in groups I and II were included in the study if their current jobs required them to be exposed regularly to radioiodine or X-ray. They were working 6 hours per day for 6 days per week in two rotating shifts. None of them received chemotherapeutic drugs or subjected to ionizing radiation for diagnostic or therapeutic purposes in the 6 months before blood collection. The annual accumulated effective dose was measured during the person’s entire working time using personal pocket dosimeters. Participants who met the inclusion criteria were included in the study. Exclusion criteria included participants in either group who had a history or confirmed diagnosis of thyroid cancer, hypothyroidism, hyperthyroidism, or thyroid parenchymal disease. Pregnant women and smokers were also not included in the study.

### Blood samples collection

One venous blood sample was collected from radiation workers and healthy controls. The blood sample (5 ml) was allowed to clot for 10–20 min at room temperature. It was centrifuged at 2000–3000 RPM for 20 min. The supernatants were collected carefully. Serum was stored at − 80 °C until used. Thyroid hormone panel (fT3, fT4, TSH), serum anti-TPO, and Tg levels were measured using the Enzyme-Linked Immunosorbent Assay (ELISA), by the recommendations of the manufacturer (Diagnostic Automation, USA). A colorimetric approach was used to detect oxidative stress markers such as MDA, H_2_O_2_, and TAC according to manufacturers’ instructions (Bio diagnostic, Egypt).

### Thyroid ultrasonography

Thyroid ultrasonographic evaluation was performed for all subjects who participated in the study. Ultrasonography was used to determine the thyroid parenchymal echo structure, thyroid volume, and the presence of thyroid nodules in workers from three groups. The thyroid parenchymal echo structure was detected as homogeneous and heterogenous by ultrasound [18]. Thyroid volume was calculated according to this formula: TV = RL [T × W × L × CF] + LL [T × W × L × CF] and the volume of the isthmus was not included [19]. The thyroid ultrasound examinations were performed by a single radiology consultant with 22 years of experience in ultrasound (Y. A.) using SIEMENS-G40 ultrasound equipment and a 7–10 MHz (MHz) linear probe.

## Statistical analyses

The IBM SPSS software programmer version 20.0 was used to examine the data (Armonk, NY: IBM Corp). Numbers and percentages were used to describe qualitative data. The normality of the data distribution was examined by the Kolmogorov-Smirnov test. The range (minimum and maximum), mean, and standard deviation characterize quantitative data. To compare categorical variables between groups, the Chi-square test was used. The student t-test was used to compare two normally distributed quantitative data groups. The Mann-Whitney test was created to compare two groups of quantitative data that were abnormally distributed. The Spearman coefficient was used to determine a relationship between two abnormally quantitative variables. The significance of the obtained results was decided at a 5% level.

## Results

### Demographic data

Demographic data of the studied groups was illustrated in Table 1. The difference in mean age and sex status between participants.

**Table 1:** Demographic data of the studied groups

	Participants exposed to radioiodine (Group I) (*n* = 40)	Participants exposed to X-ray (Group II) (*n* = 40)	Control group (Group III) (*n* = 40)	*p*
Age (years)				
Min. – Max.	24.0–57.0	22.0–51.0	22.0–51.0	0.291
Mean ± SD.	33.91 ± 10.30	31.16 ± 7.92	30.59 ± 7.12	
Sex: n (%)				
Female	18 (45%)	14 (35%)	16 (40%)	0.321
Male	22 (55%)	26 (65%)	24 (60%)	
Working period (years)				
Min. – Max.	2–20	1–38	–	
Mean ± SD.	7.50 ± 5.46	14.83 ± 10.91		
Annual effective dose (mSv)				
Min. – Max.	0.44–2.89	0.5–3.6	–	
Mean ± SD.	1.22 ± 0.88	1.53 ± 0.91		

*p*, *p*-value for comparing each radiation workers group and control group. Student t test was used

There was no significant difference in mean age and sex between Group I or Group II with Group III (*p* = 0.291 and *p* = 0.321, respectively) (Table 1).

### Thyroid function tests

Thyroid function tests of radiation workers groups and control group were shown in Table 2. Regarding participants occupationally exposed to radioiodine (Group I), mean values of TSH and fT4 showed an insignificant difference in comparison to the control group (Group III) (2.12 ± 0.90 vs. 2.16 ± 0.73, *p* = 0.896 and 1.28 ± 0.14 vs. 1.25 ± 0.25, *p* = 0.212, respectively). Contrariwise, participants from group I had significantly higher fT3 mean values than participants from group III (3.01 ± 0.41 versus 2.76 ± 0.38, *p* = 0.047*). Meanwhile, Tg values in participants from group I were significantly lower than in participants from group III (12.86 ± 11.53 vs. 18.29 ± 11.29, *p* = 0.028). Regarding anti-TPO, their mean values were substantially higher in participants from group I than in participants from group III (35.61 ± 82.35 vs. 8.40 ± 1.26, *p* < 0.001*, respectively).

**Table 2:** Comparison between the studied groups according to the thyroid function tests

Thyroid function tests	Participants exposed to radioiodine (Group I) (*n* = 40)	Participants exposed to X-ray (Group II) (*n* = 40)	Control group (Group III) (*n* = 40)	p*	p**
TSH (μIU/ml)					
Min. – Max.	0.85–4.61	0.31–3.12	0.92–3.24	0.896	0.010*
Mean ± SD.	2.12 ± 0.90	1.80 ± 1.22	2.16 ± 0.73		
Free T3 (pg/ml)					
Min. – Max.	2.35–3.56	2.44–4.25	2.09–3.43	0.047*	< 0.001*
Mean ± SD.	3.01 ± 0.41	3.49 ± 0.53	2.76 ± 0.38		
Free T4 (ng/dl)					
Min. – Max.	0.90–1.47	0.77–1.76	0.88–1.55	0.212	0.101
Mean ± SD.	1.28 ± 0.14	1.32 ± 0.20	1.25 ± 0.25		
Thyroglobulin (ng/ml)					
Min. – Max.	0.69–43.30	0.86–37.60	4.12–49.30		
Mean ± SD.	12.86 ± 11.53	10.81 ± 8.86	18.29 ± 11.29	0.028*	0.005*
Anti-Thyroid Peroxidase Ab (IU/ml)					
Min. – Max.	5.33–397.0	5.0–249.0	6.89–11.50		
Mean ± SD.	35.61 ± 82.35	31.93 ± 57.58	8.40 ± 1.26	< 0.001*	< 0.001*

p*, *p*-value for comparing between group I and group III. p**, *p*-value for comparing between group II and group III. *: Statistically significant at *p* ≤ 0.05. Student t test was used

Regarding participants occupationally exposed to X-rays (Group II), TSH levels were significantly lower than participants from group III (1.80 ± 1.22 versus 2.16 ± 0.73, *p* = 0.010* respectively). Contrariwise, fT4 showed an insignificant difference in participants of Group II in comparison to participants from group III (1.32 ± 0.20 vs. 1.25 ± 0.25, *p* = 0.101 respectively). Participants from group II had significantly higher mean fT3 values than participants from group III (3.49 ± 0.53 versus 2.76 ± 0.38, *p* < 0.001* respectively). Meanwhile, the mean serum Tg levels in participants from group II were significantly lower than in the participants from group III (10.81 ± 8.86 vs. 18.29 ± 11.29, *p* = 0.005* respectively). Regarding anti-TPO, their mean values were substantially higher in participants from group II than in the participants from group III (31.93 ± 57.58 vs. 8.40 ± 1.26, *P* < 0.001* respectively) (Table 2).

### Oxidative stress markers

The mean values of MDA and H_2_O_2_ levels were significantly higher in radioiodine-exposed workers (Group I), than in the participants from group III (5.76 ± 7.09 vs. 1.14 ± 0.67, *p* = 0.006* and 1.36 ± 0.40 vs. 0.48 ± 0.23, *p* < 0.001*, respectively). However, the mean values of TAC were significantly lower in participants from group I than in the participants from group III (0.74 ± 0.56 vs. 1.59 ± 0.51, *p* < 0.001*). For participants from group II exposed to X-ray, the mean values of MDA and H_2_O_2_ levels were significantly higher than in the participants from group III (4.04 ± 2.32 vs. 1.14 ± 0.67, *p* < 0.001* and 1.25 ± 0.39 vs. 0.48 ± 0.23, *p* < 0.001*, respectively). However, compared to the control group, the mean values of TAC were significantly lower (0.56 ± 0.23 vs. 1.59 ± 0.51, *p* < 0.001*) (Table 3).

**Table 3:** Comparison between the studied groups according to oxidative stress markers

	Participants exposed to radioiodine (Group I) (*n* = 40)	Participants exposed to X-ray (Group II) (*n* = 40)	Control group (Group III) (*n* = 40)	p*	p**
MDA (nmol/ml)					
Min. – Max.	0.13–21.84	1.52–11.10	0.26–2.50	0.006*	< 0.001*
Mean ± SD.	5.76 ± 7.09	4.04 ± 2.32	1.14 ± 0.67		
H_2_O_2_ (mM/L)					
Min. – Max.	1.01–2.42	1.20–2.34	0.16–0.93	< 0.001*	< 0.001*
Mean ± SD.	1.36 ± 0.40	1.25 ± 0.39	0.48 ± 0.23		
TAC (mM/L)					
Min. – Max.	0.08–1.74	0.19–0.94	1.08–2.71	< 0.001*	< 0.001*
Mean ± SD.	0.74 ± 0.56	0.56 ± 0.23	1.59 ± 0.51		

p*, *p*-value for comparing between group I and group III. p**, *p*-value for comparing between group II and group III. *: Statistically significant at *p* ≤ 0.05. Student t test and Mann Whitney test were used

### Thyroid ultrasonography

**Fig. 1:**
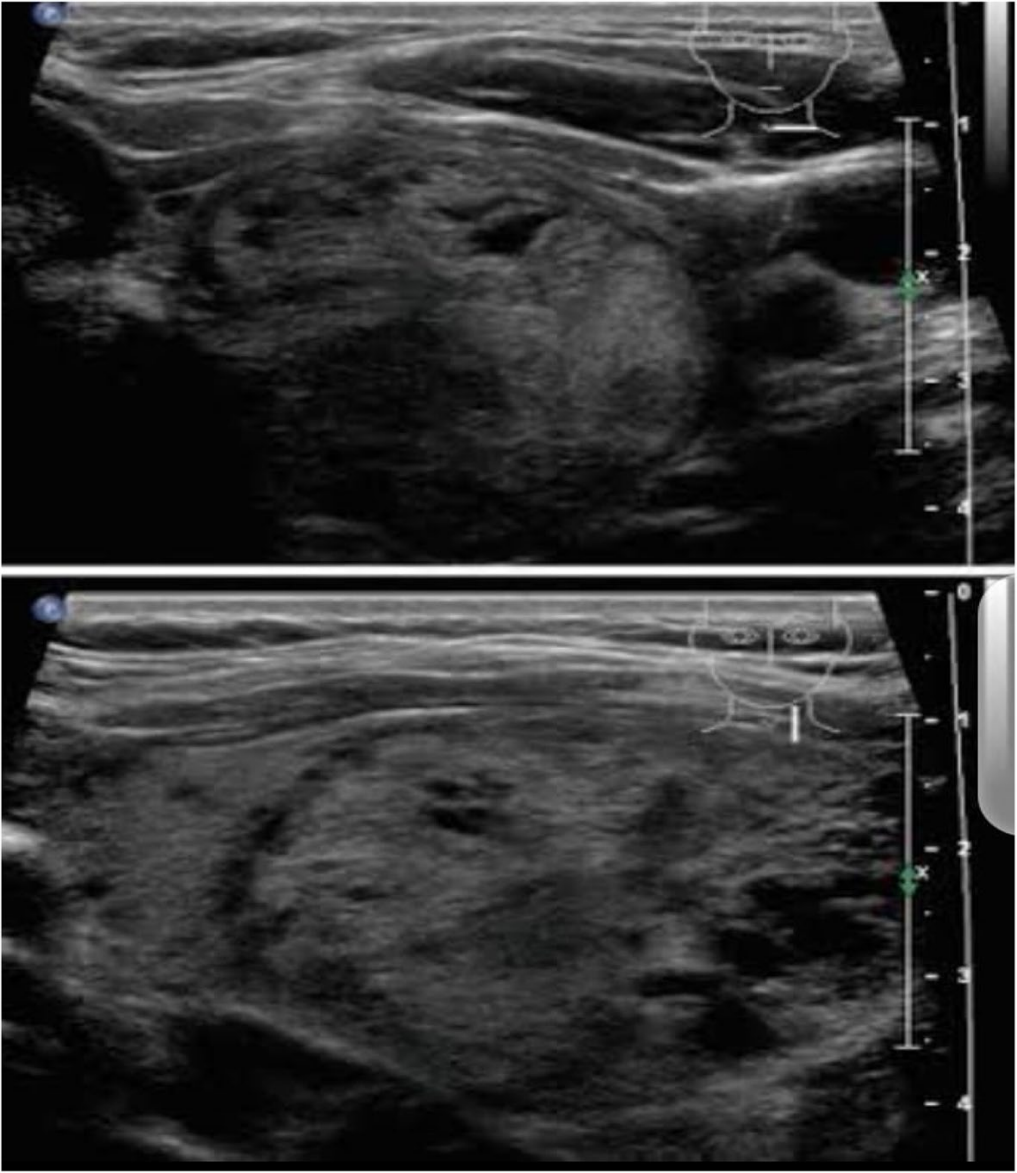
Gray-scale ultrasound images show a right thyroid nodule described as TIRADS IV in a Group I participant

**Fig. 2:**
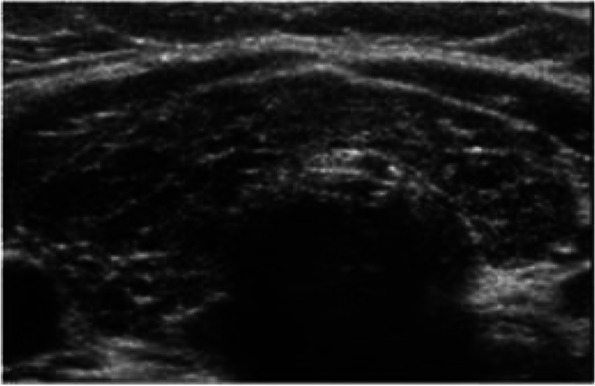
Gray-scale ultrasound images show heterogeneous thyroid parenchyma in a Group II participant

**Fig. 3:**
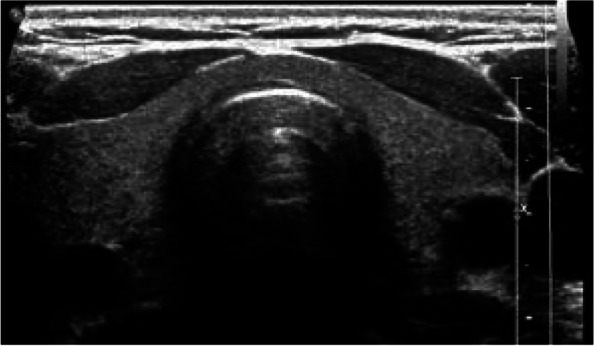
Gray-scale ultrasound images show homogeneous thyroid parenchyma in a Group III participant

The mean thyroid volume (ml) was significantly larger in participants from group I, in comparison to participants from group III (10.32 ± 3.42 vs. 4.62 ± 1.13, *p* < 0.001*). The thyroid nodule percentage in Group I was significantly higher than in the control group (*p* = 0.005*). An insignificant difference was found between the two studied groups regarding the disease rate of diffuse thyroid parenchymal disease (*p* = 0.172). The mean thyroid volume (ml) was significantly larger in participants from group II, in comparison to participants from group III (11.65 ± 5.95 vs. 4.62 ± 1.13, *p* < 0.001*). The percentage of thyroid nodules was significantly higher in participants from group II than in participants from group III (*p*= =0.021*). Moreover, the rate of diffuse thyroid parenchymal disease among participants from group II was considerably higher (*p* < 0.001*) (Table 4 and Figs. 1, 2, and 3).

**Table 4:** Comparison between the studied groups according to Thyroid Ultrasonography

	Participants exposed to radioiodine (Group I) (*n* = 40)	Participants exposed to X-ray (Group II) (*n* = 40)	Control group (Group III) (*n* = 40)	p*	p**
Thyroid volume (ml)					
Min. – Max.	7.0–18.0	4.50–28.20	3.0–8.0	< 0.001*	< 0.001*
Mean ± SD.	10.32 ± 3.42	11.65 ± 5.95	4.62 ± 1.13		
Thyroid nodules					
Normal	27 (67.5%)	26 (65%)	36 (90%)	0.005*	0.021*
Cystic	7 (17.5%)	4 (10%)	3 (7.5%)		
Solid	4 (10%)	6 (15%)	1 (2.5%)		
Mixed	2 (5%)	4 (10%)	0 (0.0%)		
% of Diffuse thyroid parenchymal disease	0%	30%	0%	0.172	< 0.001*

p*, *p*-value for comparing between group I and group III. p**, *p*-value for comparing between group II and group III. *: Statistically significant at *p* ≤ 0.05. Student t test and Chi square test were used

### Correlation between working period and annual effective dose with studied biomarkers in radiation workers

MDA levels and working period were significantly correlated (*p* = 0.013*) in participants from group I. An insignificant difference between the working period and annual dose with other studied biomarkers is illustrated in Table 5.

**Table 5:** Correlation between working period and annual absorbed dose with different studied biomarkers in radiation workers

Participants exposed to radioiodine (Group I) (*n* = 40)	Working period (years)		Annual effective dose (mSv)		Participants exposed to X-ray (Group II) (*n* = 40)	Working period (years)		Annual effective dose (mSv)	
	r_s_	p	r_s_	p		r_s_	p	r_s_	p
**TSH**	0.249	0.263	0.112	0.618	**TSH**	−0.075	0.693	0.085	0.657
**Free T3**	0.002	0.992	−0.123	0.584	**Free T3**	0.108	0.569	0.267	0.154
**Free T4**	0.257	0.248	−0.085	0.708	**Free T4**	0.226	0.230	0.176	0.352
**Thyroglobulin**	0.166	0.461	0.130	0.564	**Thyroglobulin**	0.293	0.611	−0.145	0.445
**Thyroid-Anti-peroxidase Ab**	0.047	0.837	0.310	0.098	**Thyroid-Anti-peroxidase Ab**	0.137	0.469	0.238	0.205
**MDA**	0.520	0.013^*^	0.320	0.147	**MDA**	0.121	0.526	0.344	0.062
**H**_**2**_**O**_**2**_	0.008	0.972	−0.084	0.711	**H**_**2**_**O**_**2**_	0.141	0.457	0.258	0.169
**TAC**	0.170	0.449	0.173	0.441	**TAC**	−0.005	0.980	−0.137	0.470

r_s_: Spearman coefficient*: Statistically significant at *p* ≤ 0.05

## Discussion

Several studies have established ionizing radiation’s effects on the thyroid, particularly as a significant reason for thyroid carcinoma and nodules [20–22]. The severity of the disorders linked to radiation dose has led to the conclusion that acute radiation exposure is more damaging than chronic radiation exposure. Ionizing radiation at high doses has undeniable detrimental consequences involving cancer induction. Although low-dose radiation risk is substantial due to its linkages to cancer screening tests and occupational radiation exposure, the situation is less evident at very low radiation doses [23]. The current study revealed that the mean TSH levels in radiation workers, especially exposed to X-rays, were considerably lower than in healthy controls. Contrariwise, radiation workers had a significant increase in the mean fT3 values than in the control group. Regarding ultrasonography imaging, radiation workers had a greater thyroid volume (ml), diffuse thyroid parenchymal disease, and an increased percentage of thyroid nodules. These findings point to hyperthyroidism because of occupational ionizing radiation exposure. Oxidative stress and the generation of reactive oxygen species (ROS) due to ionizing radiation exposure during work shifts may be implicated in these changes induced in the thyroid gland.

Our results were in agreement with Alawneh K et al. [24] who revealed that thyroid hormone levels might be elevated due to radiation exposure. On the contrary to our results, Wong YS et al. [25] concluded that despite low exposure doses, occupational exposure to IR in medical workers still may be linked with the declines in the serum levels of T3 and T4. In a previous study, the authors suggested that occupationally exposed medical personnel to IR have iodine deficiency and higher thyroid nodules [26]. Furthermore, Chen et al. conducted a study [27] which assumed that thyroid disorder among radiologists might be considered related to other risk factors, including working night shifts and being under heavy work stress.

A recent study conducted by Guo et al., [28] showed that T3 and T4 levels in the participants decreased slightly but significantly during the follow-up years. This study agrees in part with our results, in which T3 levels increased as the radiation dose increased, implying the existence of a dose threshold above which T3 synthesis and secretion are promoted. This study, however, found no link between radiation doses and thyroid hormone level decreases, which is consistent with the current research. This may be due to the negative feedback regulation mechanism of the thyroid system. The dynamic equilibrium of TSH, T3, and T4 levels can be maintained and the relative stability of thyroid hormone secretion can be controlled through the hypothalamus-pituitary-thyroid regulation loop which enhances TSH production to support T4 and T3 secretion when the serum T4 concentration and T3 are diminished [28].

We explain that occupational exposure, especially to radioiodine, may result in radioiodine accumulation in the thyroid. Radioiodine results in a significant β-decay component. Contrariwise, for nuclear medical personnel, γ-emission represents the primary source of external exposure [29]. Radioiodine emitted radiation directly induces DNA damage or generates reactive oxygen species (ROS). The thyroid tissue has a high concentration of NADPH oxidases (NOX), specialized ROS-generating enzymes defined as NOX. Radiation exposure increases NOX1 expression, resulting in significant ROS production in the thyroid gland after radiation exposure, demonstrating its high sensitivity to radiation. This DNA damage includes single-strand or double-strand breaks that will result in chromosomal aberrations [5, 30]. The International Commission on Radiation Protection (ICRP) and the National Council on Radiation Protection and Measurements (NCRP) have set a yearly exposure limit and preventive advice against overexposure. On the other hand, medical personnel take thyroid protection shields lightly and do not take them seriously, according to our observations in everyday practice. Staff not wearing thyroid shield are currently being exposed to ionizing radiation on a regular basis and the thyroid gland is more vulnerable to harmful effects of ionizing radiation [24]. The present study viewed that serum anti-TPO levels in radiation workers were significantly higher than in the control group. Meanwhile, serum Tg levels in radiation workers were considerably lower than in the control group. Suggesting autoimmune thyroid disease (AITD) induced by exposure to radioiodine or X-ray during work shifts.

A recent study by Albehairy A et al. [31] is constant with the present study, indicating that working personnel in the radiation field are positive for anti-TPO. In autoimmune thyroid disorders, thyroid autoantibodies such as a thyroid-stimulating antibody, anti-thyroglobulin antibody, and anti-thyroperoxidase antibody can be found. The latter is a sensitive method for detecting early subclinical autoimmune thyroid illnesses, monitoring immunotherapy response, and identifying autoimmune thyroid disease at-risk cases. The iodination of tyrosine residues in the thyroglobulin molecule is carried out by thyroid peroxidase. Anti-TPO antibodies are inductors of oxidative stress and mediate thyroid cell death in vitro [32–34].

The current study revealed that MDA and H_2_O_2_ levels increased substantially more in radiation workers than in the control group while TAC levels are decreased, reflecting that chronic low dose ionizing radiation can cause systemic oxidative stress. In addition, ionizing radiation exposure at work alters the redox status. These findings agree with previous reports [35, 36]. It has been demonstrated that ionizing radiation causes the immediate generation of ROS in eukaryotic cells via the radiolysis of water, which is an indirect consequence of radiation. This rapid increase in ROS causes oxidative stress damage to biological macromolecules such as lipids, proteins, and DNA. Radiation-induced ROS include O2•, hydrogen peroxide (H_2_O_2_), and the hydroxyl radical (OH•). Enzymatic and non-enzymatic detoxify ROS and protect cells from oxidative damage. The decrease in TAC could be attributed to the consumption of endogenous antioxidants because of free radical generation after radiation exposure [37]. In summary, professionals who work in a job that exposes them to radiation regularly should follow the recommendations of radiation protection, which include worker radiation safety education, dose monitoring of radiation, and the use of all protective shielding devices. Moreover, radiation exposure should be kept to a minimum (ALARA).

The limitation of this study is the different exposure times between participants from group I and group II which most likely affected the results of the study. Also this was a single-center study, further studies are needed.

## Conclusion

Occupational ionizing radiation exposure impacts the thyroid hormone panel and increased the risk of autoimmune thyroid disease, even at low doses. Biological monitoring of thyroid hormones and anti-TPO levels detects early affection of the thyroid gland among radiation-exposed workers.

## Data Availability

The datasets used and/or analyzed during the current study are not publicly available but available from the corresponding author on reasonable request.

## Abbreviations

AITD: Autoimmune Thyroid Disease
Anti-TPO: Anti-thyroperoxidase antibodies
ELISA: Enzyme-Linked Immunoassay
fT3: Free Triiodothyronine
fT4: Free Thyroxine
H_2_O_2_: Hydrogen Peroxide
ICRP: International Commission on Radiation Protection
IR: Ionizing Radiation
MDA: Malondialdehyde
MHZ: Megahertz
NCRP: National Council on Radiation Protection and Measurements
ROS: Reactive Oxygen Species
TAC: Total Antioxidant Capacity
Tg: Thyroglobulin
TSH: Thyroid-stimulating hormone
